# Medicinal plants used in Gabon for prophylaxis and treatment against COVID-19-related symptoms: an ethnobotanical survey

**DOI:** 10.3389/fphar.2024.1393636

**Published:** 2024-07-05

**Authors:** Marlaine Michel Boukandou Mounanga, Annais Mezui, Ludovic Mewono, Jean Bertrand Mogangué, Sophie Aboughe Angone

**Affiliations:** ^1^ Institut de Pharmacopée et de Médecine Traditionnelle (IPHAMETRA), Centre National de la Recherche Scientifique et Technologique (CENAREST), Libreville, Gabon; ^2^ Centre Hospitalier Universitaire Mère- Enfant, Fondation Jeanne EBORI, Libreville, Gabon; ^3^ Groupe de Recherche en Immunologie 2, Microbiologie appliquée, Hygiène et Physiologie, Département des Sciences de la Vie et de la Terre-Ecole Normale Supérieure, Libreville, Gabon

**Keywords:** COVID-19, Gabon, medicinal plants, prevention, treatment, survey

## Abstract

**Background:** Gabon faced COVID-19 with more than 49,000 individuals tested positive and 307 recorded fatalities since the first reported case in 2020. A popular hypothesis is that the low rate of cases and deaths in the country was attributed to the use of medicinal plants in prevention and treatment. This study aimed to document the plants used for remedial and preventive therapies by the Gabonese population during the COVID-19 pandemic and to pinpoint specific potential plant species that merit further investigation.

**Methods:** An ethnobotanical survey involving 97 participants was conducted in Libreville. Traditional healers and medicinal plant vendors were interviewed orally using a semi-structured questionnaire sheet, while the general population responded to an online questionnaire format. Various quantitative indexes were calculated from the collected data and included the relative frequency of citation (RFC), use value (UV), informant consensus factor (ICF), relative importance (RI), and popular therapeutic use value (POPUT). One-way ANOVA and independent samples *t*-test were used for statistical analyses. *p*-values ≤0.05 were considered significant.

**Results:** The survey identified 63 plant species belonging to 35 families. Prevalent symptoms treated included fever (18%), cough (16%), fatigue (13%), and cold (12%). The demographic data highlighted that 52.58% of male subjects (*p* > 0.94) aged 31–44 years were enrolled in the survey, of which 48.45% (*p* < 0.0001) and 74.73% (*p* < 0.99) of informants had university-level education. In addition, the results indicated that a total of 66% of the informants used medicinal plants for prophylaxis (34%), for both prevention and treatment (26%), exclusively for treatment (3%), and only for prevention (3%) while suffering from COVID-19, against 34% of the participants who did not use plants for prevention or treatment. *Annickia chlorantha, Citrus* sp.*, Alstonia congensis, Zingiber officinale,* and *Carica papaya* emerged as the most commonly cited plants with the highest RFC (0.15–0.26), UV (0.47–0.75), and RI (35.72–45.46) values. Most of these plants were used either individually or in combination with others.

**Conclusion:** The survey reinforces the use of traditional medicine as a method to alleviate COVID-19 symptoms, thereby advocating for the utilization of medicinal plants in managing coronavirus infections.

## 1 Introduction

After the World Health Organization lifted the public health emergency of international concern for COVID-19, statistics indicate that approximately 689 million people tested positive for COVID-19, with approximately 7 million fatalities attributed to the disease (https://www.worldometers.info/coronavirus/). Various therapeutic approaches were explored in the management of COVID-19, including antibiotics (azithromycin), antiparasitic agents (hydroxychloroquine), antiviral medications (remdesivir), monoclonal antibodies (casirivimab), steroids (dexamethasone), and immune modulators (tocilizumab), anticoagulants, as well as oxygen therapy and other supportive measures for patients experiencing respiratory distress (Mehraeen et al., 2022; Murakami et al., 2023; Panahi et al., 2023). Other approaches such as the search for new treatments using traditional remedies were also explored, as well as vaccines (Rahman et al., 2022; Soheili et al., 2023).

Gabon, with a population of approximately 2.3 million, has grappled with COVID-19, with statistics revealing approximately 49,000 individuals testing positive and resulting in 307 recorded fatalities since the first reported case in 2020 (https://www.worldometers.info/coronavirus/country/gabon/). The diagnostic of the disease was carried out by Laboratoire Professeur Daniel Gahouma. The latter was the specialized state-of-the-art technology specifically established to address the challenges posed by the pandemic. In addition, COVID-19 treatment protocols in Gabon have involved antibiotherapy in combination with vitamins, paracetamol, zinc, and vaccine.

However, despite the availability of medications and vaccines, and due to fears regarding potential adverse effects associated with vaccination, the Gabonese population has turned to medicinal plants for the prophylaxis and treatment of symptoms associated with this viral disease, similar to the populations of several other countries including Algeria, Brazil, Colombia, Cameroon, Morocco, and Peru (Belmouhoub et al., 2021; Villena-Tejada et al., 2021; Chebaibi et al., 2022; Cordoba-Tovar et al., 2022; Mvogo Ottou et al., 2022; da Silva et al., 2023). Most Gabonese people often rely on medicinal plants due to their long tradition of plant-based medicine. The Gabonese population strongly believes in the efficacy of medicinal plants, considering them crucial in managing COVID-19, particularly due to symptoms resembling those of malaria and flu. Indeed, several symptoms such as respiratory disorders, colds, coughs, fever, and joint pain commonly associated with flu and malaria are typically treated using plant-based remedies by the population. Ancestral knowledge passed down from generation to generation may contain treasures of effective natural remedies against this virus. Therefore, the present study aimed to document and valorize plants used by the Gabonese population to prevent or cure COVID-19 and to identify specific plant species deserving further investigation as potential treatments against coronavirus infections.

## 2 Materials and methods

### 2.1 Data collection

**FIGURE 1:**
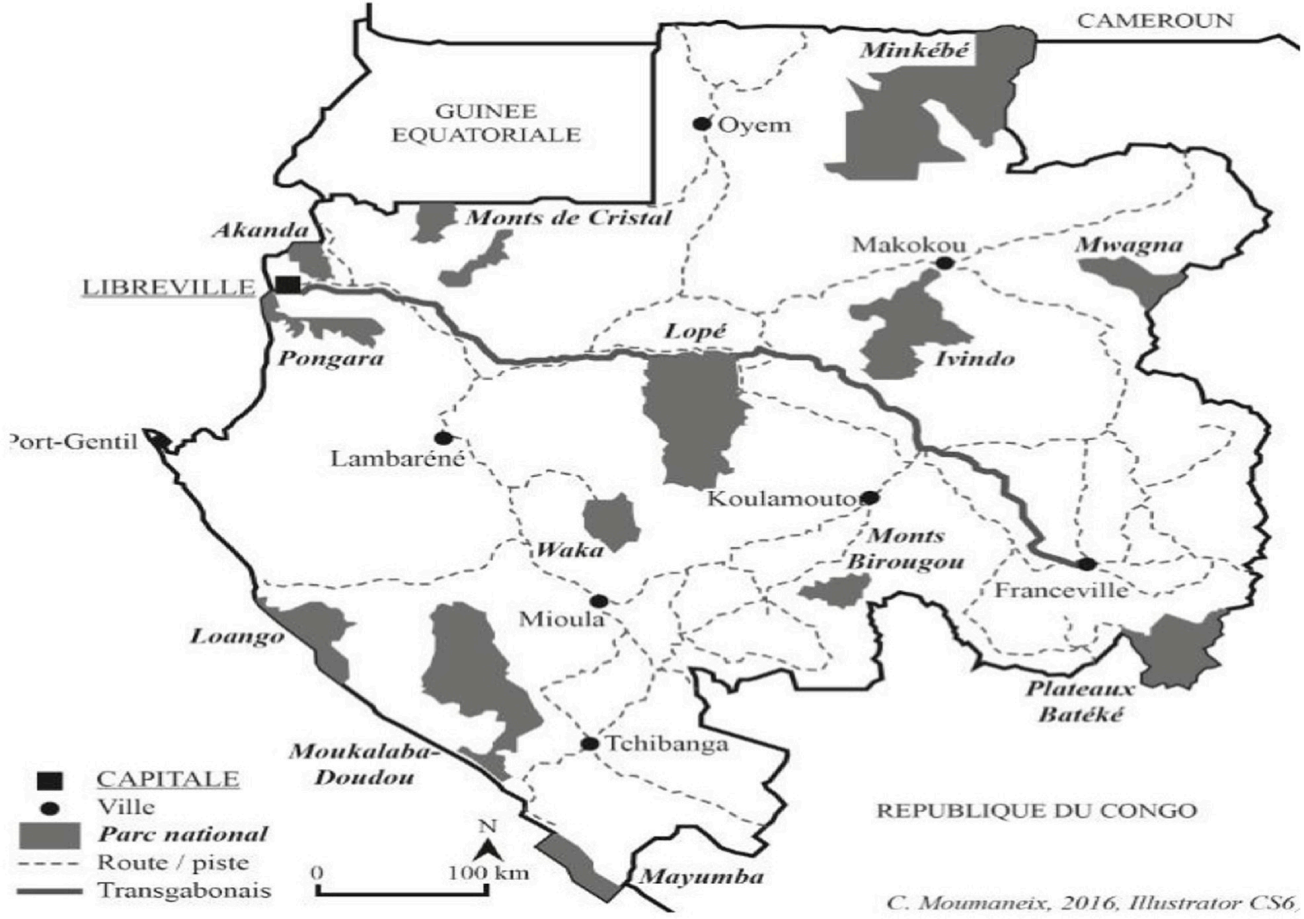
Map of Gabon (source: ANPN, s.d.).

The survey was conducted in Libreville (Figure 1) between February and June 2022 involving participants from the general population, traditional healers, and medicinal plant vendors. A semi-structured questionnaire was developed for conducting direct interviews with randomly selected traditional healers and medicinal plant vendors. Meanwhile, an online questionnaire format was employed to engage the general population. This approach aimed to collect a comprehensive range of information within the population. The questionnaire was converted into a Google Docs form using Google services and was disseminated through various online-based social media platforms such as Facebook, Instagram, Messenger, and WhatsApp. These platforms were chosen as they were perceived to be the most accessible means of reaching the maximum population regarding the governmental measures of partial lockdown and restrictions on movement. A total of 97 participants responded to the survey, comprising both online respondents and those who participated in oral interviews. The questionnaire was designed with two main sections: the first focused on gathering information about the informants (including age, sex, level of education, and ethnic group affiliation), while the second aimed to obtain details regarding medicinal plants, their forms, prescriptions, COVID status, and methods of preparation.

### 2.2 Data analysis

#### 2.2.1 Quantitative analyses of the ethnobotanical data

Various quantitative indexes were calculated from the collected data and included the relative frequency of citation (RFC), Use Value (UV), Informant Consensus Factor (ICF), Relative Importance (RI) and Popular Therapeutic Use Value (POPUT) (Anwar et al., 2023; Naceiri et al., 2021; Ndhlovu et al., 2023).

##### 2.2.1.1 Relative Frequency of Citation (RFC)

The Relative Frequency of Citation (RFC) demonstrates the local importance of each plant species. The RFC value ranges from 0 (none of the informants indicate a plant species as useful), to 1 (all informants indicate it as useful). RFC has been calculated as follows:

RFC=FC/N

Where FC denotes the number of informants mentioning the use of the species in any symptoms and N is the total number of informants participating in the survey.

##### 2.2.1.2 The use value (UV)

The UV is an index highlighting the relative importance of plants known locally in traditional medicine. The use value of a plant species varies according to its cultural, geographical, and biological context. The UV determines the most frequently indicated plants in the treatment of an ailment. Use value was calculated using the following formula:

$$\text{UV}=\frac{\sum UI\;}{N}$$

Where *UI* is number of uses recorded for a given species by each informant, and *N* is the total number of informants participating in the survey.

##### 2.2.1.3 Relative Importance (RI)

RI helps in the prioritization of plants based on their importance and prevalence in local knowledge systems. It is a useful tool for identifying significant plants with cultural, medical, or ritual significance to a society. It was determined as:

$$\text{RI}=\frac{\left(R\cdot Ph+R\cdot BS\right)\;}{2}\times 100$$

Where ‘*R·Ph*’ stands for relative pharmacological properties. ‘*R·Ph*’ is calculated by dividing number of uses (U) by total number of use reports. ‘*R·BS*’ is calculated by dividing number of diseases treated by a plant species by total number of diseases.

##### 2.2.1.4 Informant Consensus Factor (ICF)

This index was calculated for informants’ agreement on the reported treatment based on each category of disease. The following formula was used to calculate the informant consensus factor (ICF):

$$\text{ICF}=\frac{\left(Nur\;–\;Nt\right)\;}{\left(Nur\;–\;1\right)},$$

where “Nur” is the total use reports for each category and “Nt” is the total number of species used for that category. The ICF scale is 0–1. A number close to 1 implies a high level of agreement or consensus among informants regarding the relevance of a given use category and the plants associated with it. A score closer to 0 shows a lack of consensus, implying that informants may have various or varying ideas regarding the relevance of a certain use category or the plants used for that purpose.

##### 2.1.1.5 Popular therapeutic use value

The popular therapeutic use value (POPUT) shows the significance of a plant species for medicinal and therapeutic uses. The following formula was used to calculate the popular therapeutic use value:

$$\text{POPUT}=\frac{NURIT}{TUR},$$

where “NURIT” is the number of use reports for each illness or therapeutic effect and “TUR” is the total number of use reports.

#### 2.2.2 Statistical analysis

The recorded data were tabulated on Microsoft Excel spreadsheets. A descriptive and quantitative statistical method was applied (one-way ANOVA and independent samples *t*-test; *p*-values ≤0.05 were considered significant) to analyze and summarize the data.

## 3 Results

### 3.1 Sociodemographic features of informants

The social–demographic features of informants are presented in Table 1. The gender distribution revealed that women constituted 47.42% of the respondents, while men were more predominant at 52.58%. The average age of participants was 39.41 years, ranging from 18 to 66 years, with the age group of 31–44 years being the most represented at 48.45% (*p* < 0.0001). A significant majority of informants (74.73%) had attended university, while only 3.3% had completed primary school. The most represented ethnic affiliation was the Fang (30.77%), followed by the Punu (19.78%) and the Nzebi (13.19%). Ethnic groups such as the Awandji, Benga, and Haussa were among the least represented.

**TABLE 1:** Sociodemographic data about the participants of the study.

Variable	Demographic category	Number of informants (n = 97)	Frequency (%)	*p*-value
Female		46	47.42	>0.94
Male		51	52.58	
Age	18–30	20	20.62	<0.0001
	31–44	47	48.45	
	45–66	30	30.93	
Education	Primary school	3	3.09	>0.9999
	Secondary	21	21.65	
	University	73	75.26	
Ethnic group	Awandji	1	1.03	>0.9999
	Benga	1	1.03	
	Haussa	1	1.03	
	Kota	1	1.03	
	Kwélé	1	1.03	
	Vili	1	1.03	
	Vungu	1	1.03	
	Adouma	3	3.09	
	Massango	3	3.09	
	Myene	3	3.09	
	Mitsogo	8	8.25	
	Ghisir	11	11.34	
	Ndzebi	12	12.37	
	Punu	18	18.56	
	Fang	32	32.99	

### 3.2 Frequency of COVID-19-related symptoms treated

**FIGURE 2:**
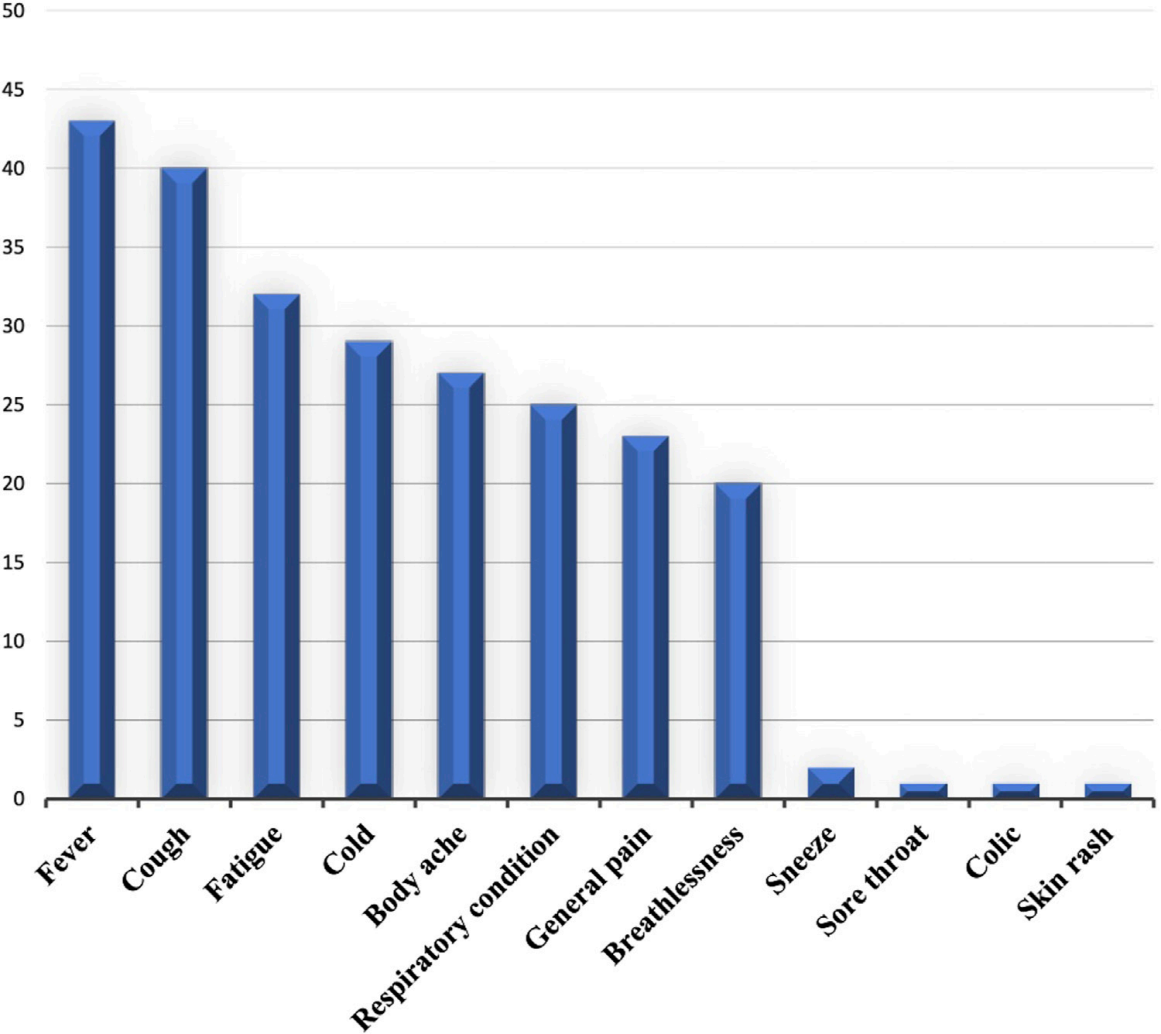
Treated symptoms.

COVID-19 symptoms are various. In this survey, prevalent symptoms included fever (18%), cough (16%), fatigue (13%), and cold (12%), as shown in Figure 2. Additionally, respiratory conditions (10%), general pain (8%), and breathlessness (7%) were managed by informants to a lesser extent. Sneeze, sore throat, and colic associated with COVID-19 were cited at 2% and 1%.

### 3.3 Frequency of plant parts used

**FIGURE 3:**
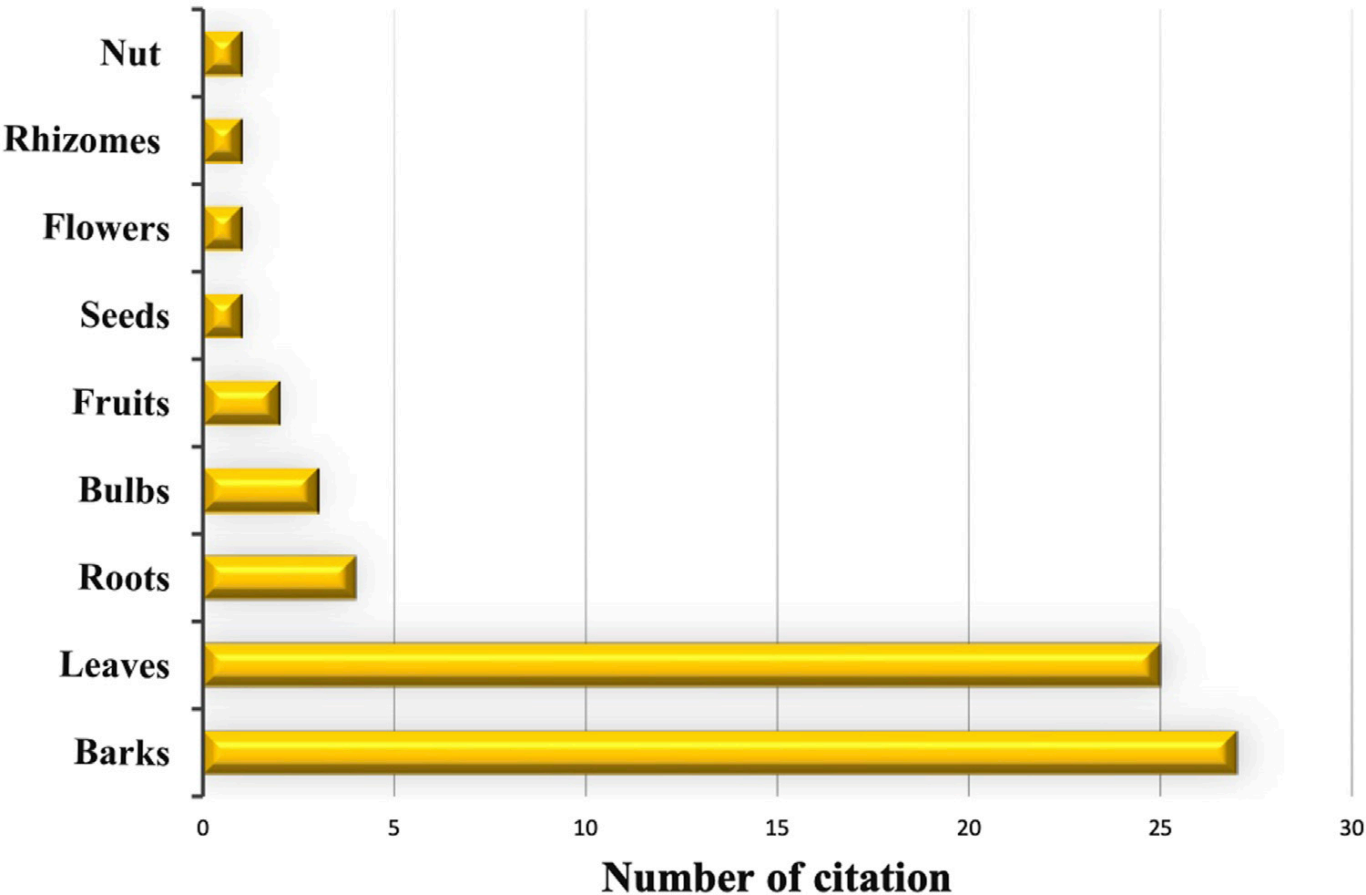
Plant parts used.

The survey unveiled that nearly all plant parts were used in the treatment of COVID-19. Informants mostly used bark (42%) and leaves (38%) for their remedies, while roots, bulbs, and fruits were used to a lesser extent, accounting for 7%, 6%, and 3%, respectively. Less commonly utilized were parts such as seeds, flowers, rhizomes, and nuts, each representing 1% (Figure 3).

### 3.4 Frequency of the methods of preparation and administration of ethnomedicinal remedies

**FIGURE 4:**
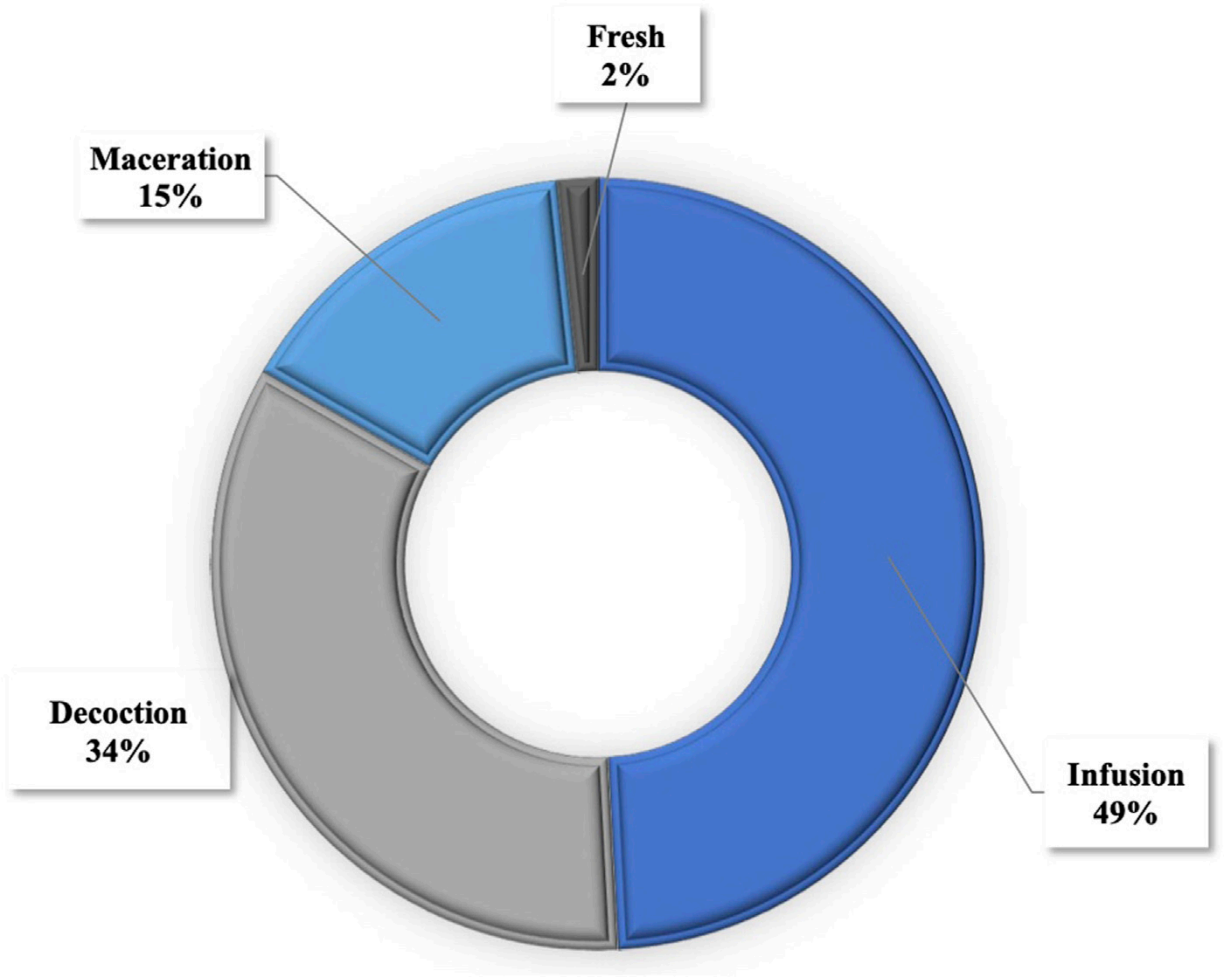
Mode of preparation.

**FIGURE 5:**
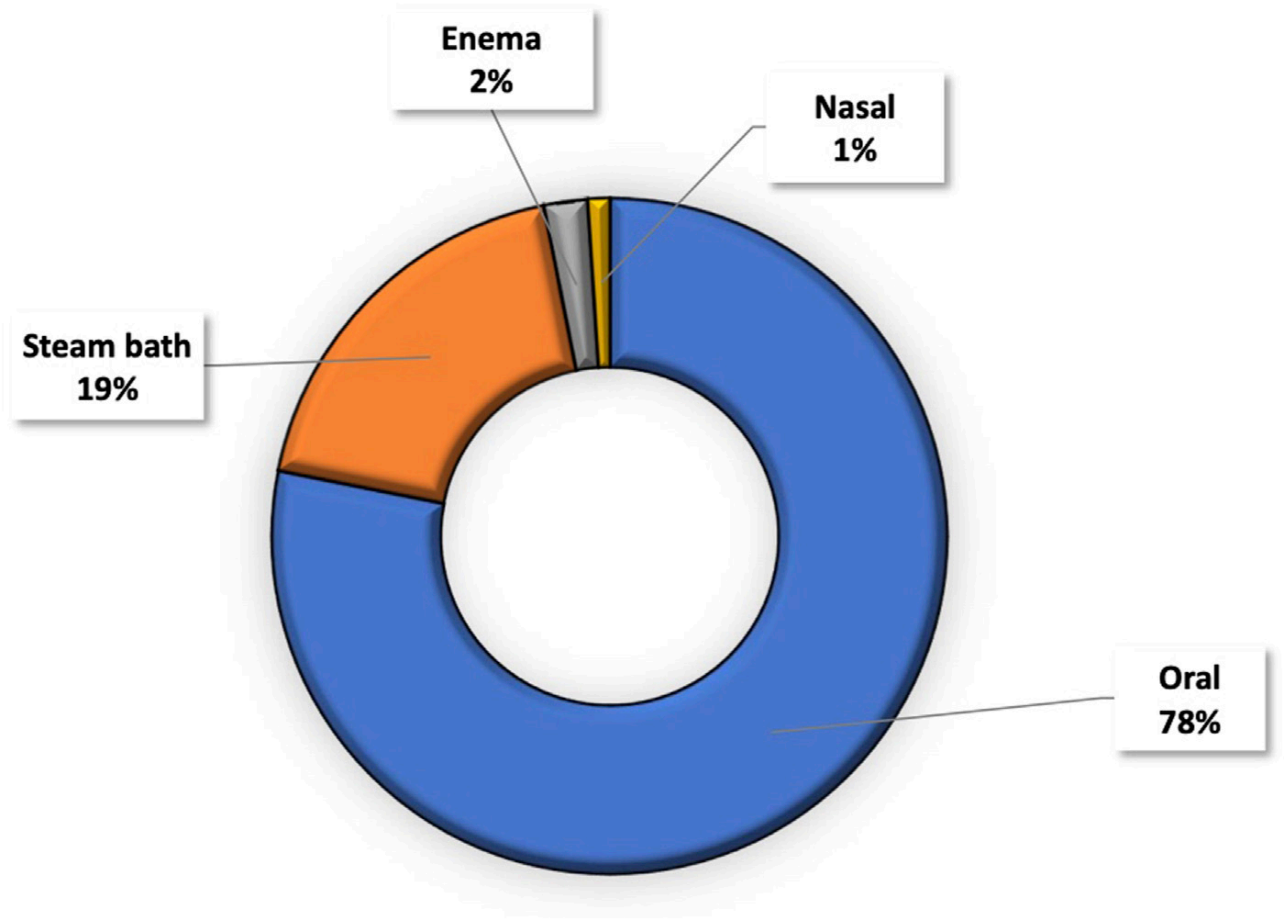
Mode of administration.

In the formulation of plant-based remedies for the prevention and treatment of COVID-19, methods such as infusion, decoction, and maceration were predominantly utilized, comprising 49%, 34%, and 15%, respectively (Figure 4). The administration of these remedies was primarily oral (78%), while 19% were applied through steam baths. Enema and nasal administration accounted for 2% and 1%, respectively (Figure 5).

### 3.5 Frequency of the disease amongst the informants and use of plants in prevention or treatment

**FIGURE 6:**
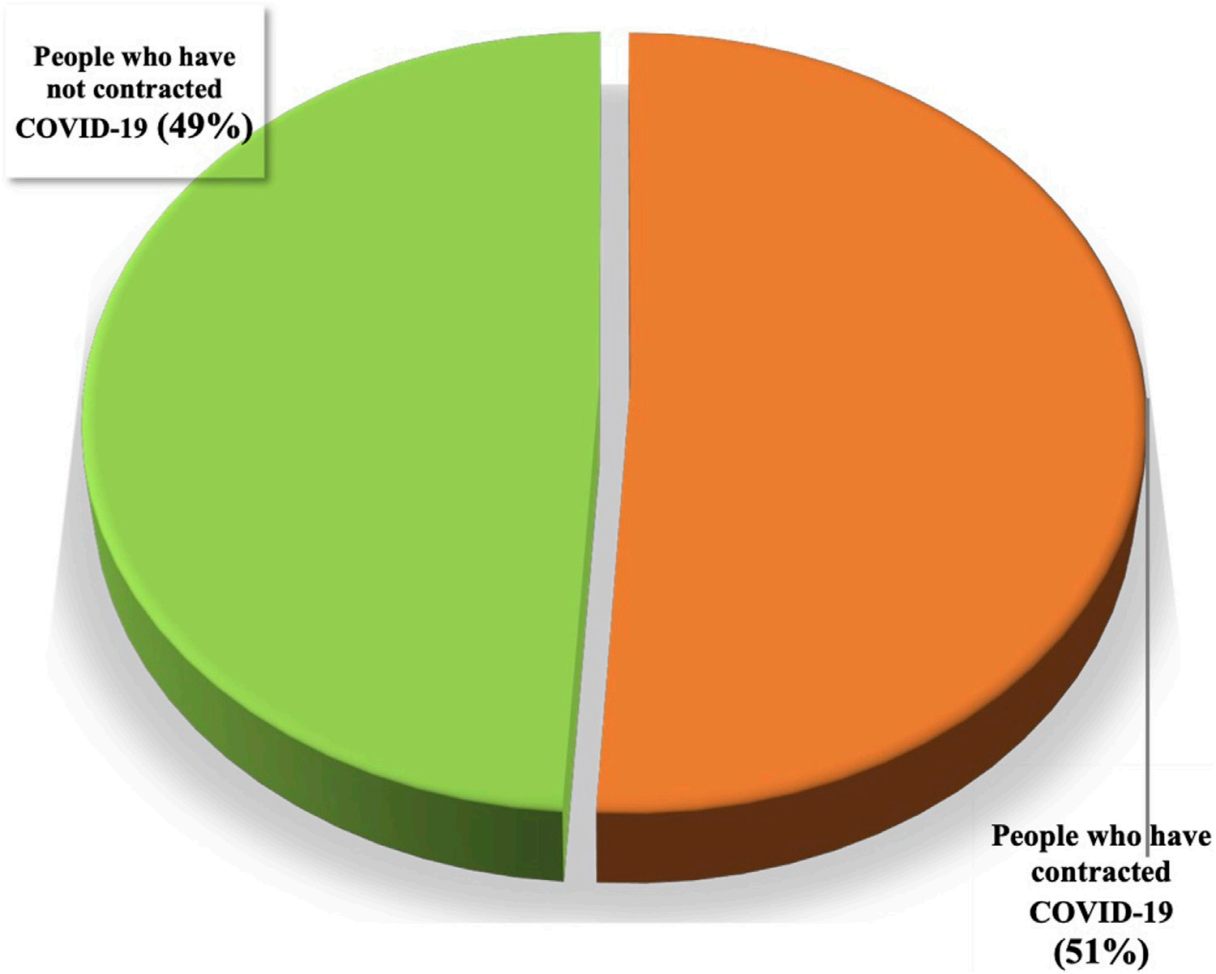
Proportion of people who have contracted COVID-19.

**FIGURE 7:**
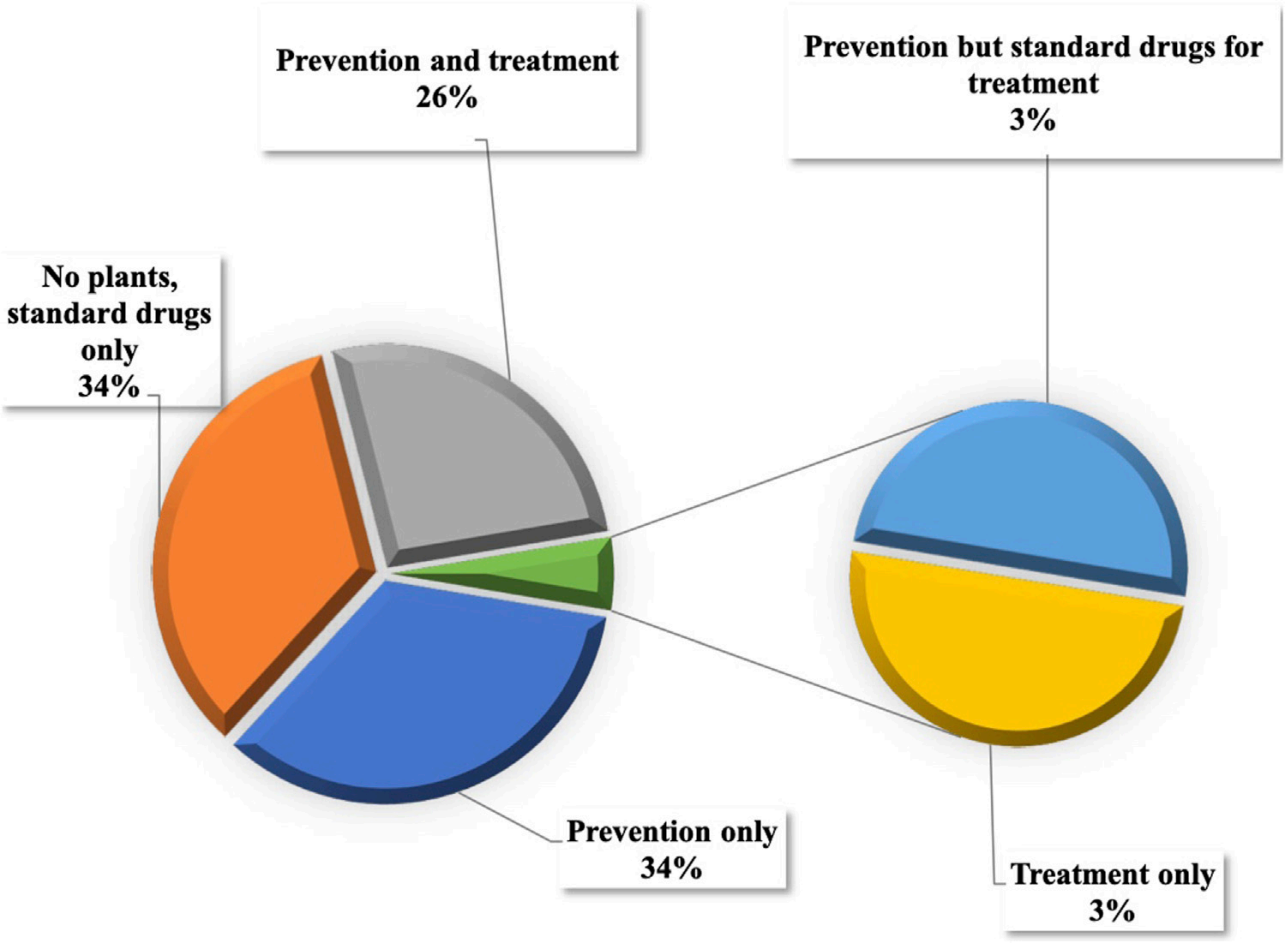
Use of medicinal plants in the treatment and/or prevention of COVID-19.

Among the participants, 51% tested positive for COVID-19, as confirmed by PCR analysis, while 49% either did not exhibit any symptoms or did not contract the disease (Figure 6). The survey also uncovered that 34% of the informants did not use plants for either prevention or treatment. Among the remaining participants, 34% utilized plants for prophylaxis, 26% for both prevention and treatment, 3% exclusively for treatment, and 3% used medicinal plants solely for prevention in conjunction with standard drugs while they were sick with COVID-19 (Figure 7).

### 3.6 Ethnomedicinal plants used

The survey on medicinal plants used for the prevention and treatment of COVID-19 within the general population, traditional healers, and medicinal plant vendors identified 63 plant species belonging to 35 families (Table 2). These plants were utilized either individually or in combination with other species to formulate remedies. The listed species are presented in alphabetical order by family. For each plant, we provide information on the family, scientific name, vernacular name, part used, and growth form.

**TABLE 2:** Ethnobotanical data on plants used to treat and prevent COVID-19.

N°	Family	Scientific name	Vernacular name	Plant parts	Growth form	RFC	UV	RI (%)
1	Acanthaceae	*Acanthus montanus* (Nees) T.Anderson	Mabangue pele	Roots	Perennial	0.02	0.03	12.66
2	Anacardiaceae	*Mangifera indica* L.	Mwiba	Leaves and barks	Tree	0.11	0.32	34.94
3		*Pseudospondias longifolia* Engl.	Musungubali	Barks	Tree	0.01	0.04	16.88
4	Annonaceae	*Annona muricata* Lin.	Corossol	Leaves	Tree	0.02	0.05	21.09
5		*Annickia chlorantha* (Oliv.) Setten and Maas	Nfo’o	Barks	Tree	0.26	0.75	45.46
6		*Xylopia aethiopica* (Dunal) A. Rich	Mugana	Leaves	Tree	0.01	0.02	8.44
7		*Greenwayodendron suaveolens* Engl. and Diels	Muamba noir	Leaves and barks	Tree	0.12	0.3	18.17
8		*Cleistopholis staudtii* Engl. and Diels	Ovoc	Barks	Tree	0.01	0.04	16.88
9	Apocynaceae	*Alstonia congensis* Engl.	Ekouk	Leaves and barks	Tree	0.22	0.71	45.25
10		*Tabernanthe iboga* Baill.	Dibuga	Root barks	Shrub	0.02	0.02	8.44
11		*Picralima nitida* T. Durand and H. Durand	Dugundu	Barks and fresh leaves	Tree	0.06	0.13	21.5
12	Asteraceae	*Ageratum conyzoides* L.	Burongu	Barks	Weed	0.01	0.04	16.88
13		*Artemisia annua* L.		Leaves	Shrub	0.1	0.25	34.58
14		*Gymnanthemum amygdalinum *(Delile) Sch.Bip. syn *Vernonia amygdalina*	Ndole	Leaves	Shrub	0.06	0.23	38.64
15	Bignoniaceae	*Newbouldia laevis* (P.Beauv.) Seem. ex Bureau	Isope	Leaves and barks	Tree	0.08	0.24	34.53
16	Bombacaceae	*Ceiba pentandra* (L.) Gaertn.	Dum	Leaves	Tree	0.01	0.04	16.88
17	Burseraceae	*Aucoumea klaineana* Pierre	Okume	Barks	Tree	0.08	0.3	18.17
18		*Canarium schweinfurthii* Engl.	Aiele	Barks	Tree	0.01	0.02	8.44
19		*Dacryodes edulis* (G.Don.) H. J. Lam.	Safu	Barks and leaves	Tree	0.01	0.05	16.93
20	Caricaceae	*Carica papaya* L.	Mulolu	Leaves	Tree	0.15	0.62	44.78
21	Combretaceae	*Combretum micranthum* G.Don	Kinkeliba	Roots	Shrub	0.08	0.35	35.1
22	Costaceae	*Costus lucanusianus* JA.Braun. and K.Schum.	Mikwissa	Leaves	Perennial herb	0.05	0.21	34.37
23	Euphorbiaceae.	*Alchornea cordifolia* (Schumach) Müll. Arg.	Mumbundjeni	Leaves	Shrub or small tree	0.01	0.03	12.66
24	Fabaceae	*Cylicodiscus gabunensis* Harms	Madume	Leaves	Tree	0.02	0.08	17.08
25		*Copaifera religiosa* J.Léonard	Mutombi	Barks	Tree	0.01	0.01	4.22
26		*Distemonanthus benthamianus* Baill.	Muvengui	Barks	Tree	0.02	0.05	12.76
27		*Osodendron altissimum* (Hook.f.) E.J.M.Koenen	Difyoru balossi	Barks	Tree	0.01	0.01	4.22
28		*Pterocarpus soyauxii* Taub.	Padouk	Barks	Tree	0.03	0.1	17.19
29		*Pentaclethra macrophylla* Benth.	Muvandji	Barks	Tree	0.02	0.04	16.88
30		*Senna occidentalis* (L.) Link	Muwiwisi	Leaves	Shrub	0.02	0.04	16.87
31		*Tetrapleura tetraptera* (Schum. and Thonn.) Taub.	Gyaga	Fruit	Tree	0.03	0.06	25.31
32		*Guibourtia tessmannii* (Harms) J.Léonard.	Kevazingo	Barks	Tree	0.03	0.1	17.19
33	Hypericaceae	*Harungana madagascariensis* Lam. ex Poiret	Musasa	Barks	Tree	0.01	0.02	8.44
34	Irvingiaceae	*Irvingia gabonensis* (Aubry-Lecomte ex O’Rorke) Baill.	Mundjuka	Barks	Tree	0.01	0.04	16.88
35	Lamiaceae	*Mentha suaveolens* Ehrh.		Leaves	Perennial herb	0.03	0.07	17.03
36		*Ocimum gratissimum* L.	Messep	Leaves	Perennial herb	0.07	0.2	42.65
37	Lauraceae	*Persea americana* Mill.	Muvoka	Leaves	Tree	0.01	0.02	8.44
38	Lecythidaceae	*Petersianthus macrocarpus* (P.Beauv.) Liben	Mbindju	Ecorces	Tree	0.01	0.01	4.22
39	Liliaceae	*Allium cepa* L.	Oignon	Bulbs	Perennial herb	0.02	0.03	12.66
40		*Allium sativum* L.	Ail	Bulbs	Perennial herb	0.04	0.1	25.52
41		*Aloe vera* (L.) Burm.f.	Aloe	Leaves	Perennial herb	0.01	0.07	29.53
42	Malvaceae	*Cola nitida* (Vent.) Schott and Endl.	Cola	Nut and barks	Tree	0.05	0.14	25.73
43	Moraceae	*Milicia excelsa* (Welw.) C.C.Berg.	Iroko	Barks	Tree	0.02	0.02	8.44
44	Moringaceae	*Moringa oleifera* Lam.	Moringa	Leaves	Tree	0.04	0.09	21.3
45	Musaceae	*Musa x paradisiaca* L.	Mupala	Leaves	Perennial	0.11	0.26	34.63
46	Myristicaceae	*Pycnanthus angolensis* (Welw.) Warb.	Ilomba	Barks	Tree	0.01	0.01	4.22
47		*Scyphocephalium mannii* (Benth.) Warb.	Sorro	Barks	Tree	0.01	0.07	17.04
48		*Staudtia kamerunensis* Warb.	Niove	Barks	Tree	0.09	0.45	18.95
49	Myrtaceae	*Psidium guajava* L.	Ngwaba	Leaves	Shrub or small tree	0.07	0.2	34.32
50		*Syzygium aromaticum* (L.) Merr. and L.M.Perry.	Clove	Dried flowers	Tree	0.04	0.08	29.58
51	Poaceae	*Cymbopogon citratus* (DC.) Stapf	Esosi	Leaves	Perennial grass	0.1	0.26	34.63
52	Rhamnaceae	*Maesopsis eminii* Engl.	Mosangea	Barks	Tree	0.01	0.01	4.22
53	Rubiaceae	*Mitragyna ciliata* Aubrév. and Pellegr.	Bahia	Barks	Tree	0.09	0.13	17.34
54		*Coffea mannii* (Hook.f.) A.P.Davis	Azeme	Barks	Shrub	0.01	0.02	8.44
55		*Sarcocephalus pobeguinii* Hua ex Pobég. and Pellegr.	Kombe ningo	Barks	Tree	0.01	0.07	17.04
56	Rutaceae	*Citrus* sp.	Diali	Fruits, barks, leaves	Shrubs or small tree	0.23	0.66	36.66
57		*Zanthoxylum heitzii* (Aubrév. and Pellegr.) P. G. Waterman	Ndungu	Leaves and barks	Tree	0.01	0.01	4.22
58	Simabouraceae	*Simaba africana* Baill.	Issindu ighale	Roots	Tree	0.03	0.03	12.66
59	Solanaceae	*Capsicum chinense* Jacq.	Nungu	Seeds	Shrub	0.02	0.04	16.88
60	Tiliaceae	*Ancistrocarpus densispinosus* Oliv.	Eege	Leaves	Shrub or small tree	0.01	0.04	16.88
61	Urticaceae	*Musanga cecropioides* R.Br. ex Tedlie	Parasolier	Barks	Tree	0.03	0.13	17.34
62	Vitaceae	*Cissus quadrangularis* L.	Dyaba	Aerial parts	Perennial herb	0.02	0.08	17.08
63	Zingiberaceae	*Zingiber officinale* Roscoe	Maketa	Rhizomes	Perennial herb	0.17	0.47	35.72

The family most abundantly represented, with the highest number of species, was Fabaceae (nine species), followed by Annonaceae (five species), Apocynaceae, Asteraceae, Burseraceae, Liliaceae, Myristicaceae, and Rubiaceae (three species each). Other families were represented by only one or two species, such as Lamiaceae, Musaceae, and Zingiberaceae. These plants were primarily characterized as trees, perennials, shrubs, or weeds. Table 3 presents 10 popular recipes cited by the informants for either prevention, treatment, or both. The table indicates the recipe, the plant parts used, the modes of preparation and administration, and the posology, which was mostly 2–3 times per day, especially for the remedies taken orally. The recipes were a mixture of at least two different plants and could be made of more than nine plants.

**TABLE 3:** Ten popular recipes used in the prevention and/or treatment of COVID-19-related symptoms.

	Recipe		Plant parts	Preparation	Posology	Administration
1	*Annickia chlorantha + Alstonia congensis + Syzygium aromaticum*	Nfo’o + Ekouk + Clous de Girofle	Barks and flower	Maceration, infusion, and decoction	2–3 times/day	Drink
2	*Musa x paradisiaca + Carica papaya + Mangifera indica + Cymbopogon citratus + Citrus* sp. *+ Aloe vera + Psidium guajava*	Mupala + Mulolu + Mwiba + Esosi + Diali + Aloe+ Ngwaba	Dead leaves, fresh leaves, and fruit	Decoction	2–3 times/day	Drink
3	*Mangifera indica + Psidium guajava + Cymbopogon citratus*	Mwiba + Ngwaba + Esosi	Leaves	Decoction	Twice/day	Drink Steam bath
4	*Annickia chlorantha + Carica papaya + Zingiber officinale + Citrus* sp.	Nfo’o + Mulolu + Maketa + Diali	Barks, fruits, rhizome, and leaves	Decoction	2–3 times/day	Drink
5	*Allium sativum + Citrus* sp. *+ Zingiber officinale*	Ail + Diali + Maketa + Honney	Fruit, bulb and rhizome	Maceration	More than three times/day	Drink
6	*Gymnanthemum amygdalinum + Carica papaya + Mangifera indica*	Ndolè+ Mulolu + Mwiba	Leaves	Infusion	2–3 times/day	Drink
7	*Moringa oleifera + Senna occidentalis*	Moringa + Muwiwisi	Barks and leaves	Infusion	Once/day	Steam bath
8	*Artemisia annua + Cymbopogon citratus + Annona muricata + Tetrapleura tetraptera*	Artemesia + Esosi + corosole + Gyaga	Leaves and fruit	Infusion	Once/day	Enema
9	*Annickia chlorantha + Alstonia congensis + Ocimum gratissimum*	Ńfo’o + Ekouk + Messep	Barks	Infusion	Once/day	Drink
10	*Senna occidentalis + Newbouldia laevis + Zingiber officinale + Citrus* sp. *+ Cymbopogon citratus + Allium cepa + Allium sativum +* *Syzygium aromaticum + Cola nitida*	Kinkéliba + Isope + Maketa + Diali + Esosi + Oignon + Ail + Clous de Girofle + Cola	Leaves, fruit, rhizome, and bulb	Maceration, infusion, and decoction	2–3 times/day	Drink

### 3.7 Importance of the plants through RFC, UV, and RI

Several indexes allowed the identification of the most valuable plants that were used to treat COVID-19 symptoms in Gabon (Table 2). On a global scale, species exhibiting the highest values across the various indexes calculated are deemed useful and significant for COVID-19 management and should be further assessed through pharmacological analysis for drug development purposes.

The relative frequency of citation (RFC) index varied from 0.01 to 0.26 and indicated that species like *Annickia chlorantha* (0.26), *Citrus* sp. (0.23), *Alstonia congensis* (0.22), *Zingiber officinale* (0.17), and *Carica papaya* (0.15) were the most frequently cited by informants. The lowest index calculated (0.01) was for plant species like *Ageratum conyzoides, Dacryodes edulis*, or *Sarcocephalus pobeguini.*

In the present study, the species have use values (UVs) ranging from 0.01 to 0.75. Species such as *Annickia chlorantha* (0.75), *Alstonia congensis* (0.71)*, Citrus* sp. (0.66), *Carica papaya* (0.62), *Zingiber officinale* (0.47), *Staudtia kamerunensis* (0.45), and *Mangifera indica* (0.32) presented the highest values. The lowest value of 0.01 was found for *Zanthoxylum Heitzii* and *Copaifera religiosa.*

The relative importance (RI) index had percentages ranging from 4.22% to 45.46%. The highest values were displayed by *Annickia chlorantha* (45.46%), *Alstonia congensis* (45.25%), *Carica papaya* (44.78%), *Ocimum gratissimum* (42.65%), *Gymnanthemum amygdalinum* (38.64%), *Citrus* sp. (36.66%), *Zingiber officinale* (35.72%), *Combretum micranthum* (35.1%), *Mangifera indica* (34.94%), *Cymbopogon citratus* (34.64%), *Musa x paradisiaca* (34.64%), and *Artemisia annua* (34.58%). The lowest value calculated (4.22%) corresponded to *Zanthoxylum heitzii* and *Copaifera religiosa.*

### 3.8 ICF and POPUT

**FIGURE 8:**
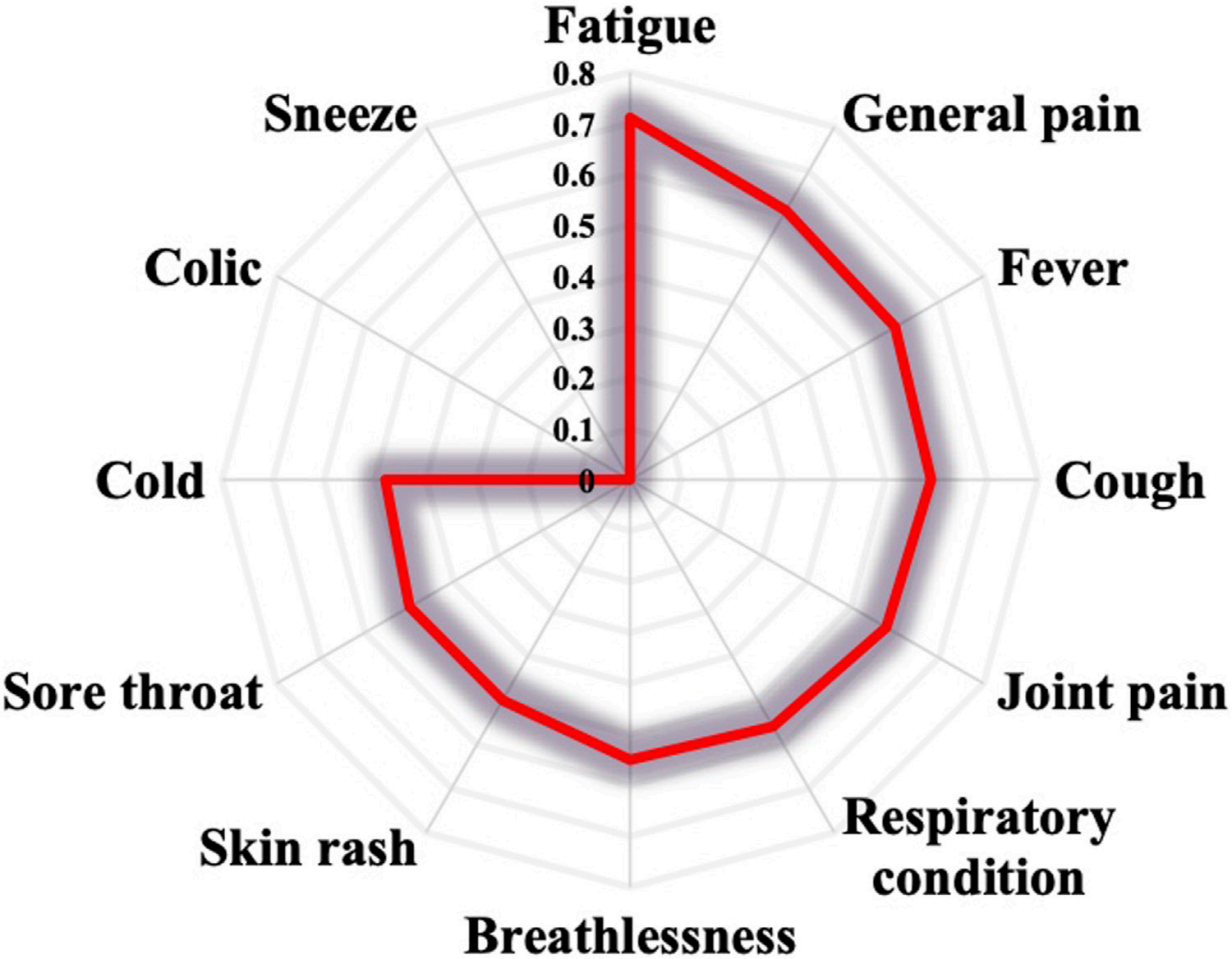
Informant consensus factor (ICF).

The survey results revealed that the cited plants were employed to address various symptoms associated with COVID-19, including cold, cough, breathlessness, fatigue, fever, and general pain. The calculated indexes indicated that ICF values ranged from 0 to 0.71. The highest ICF (0.71) was observed for fatigue, followed by general pain (0.61), fever (0.6), cough (0.59), joint pain (0.58), respiratory condition (0.56), and breathlessness (0.55). The lowest ICF values were obtained for colic and sneezing (Figure 8).

**FIGURE 9:**
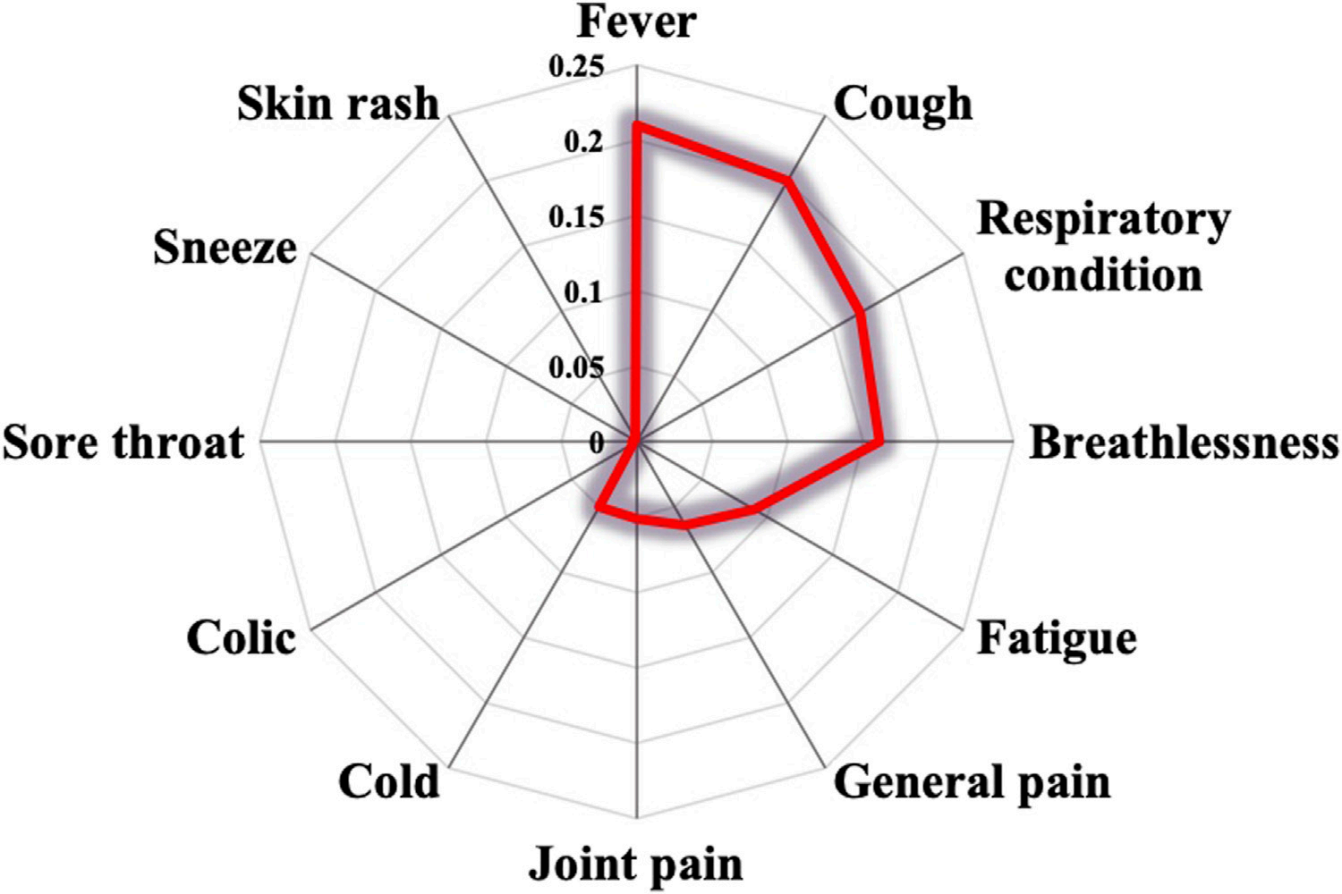
Popular therapeutic use value (POPUT).

Regarding the POPUT index, the values varied from 0.002 to 0.21. Symptoms such as cough, fever, respiratory condition, and breathlessness obtained the highest values (higher than 0.1), while the lowest values were calculated for skin rashes, colic, and sore throat (Figure 9).

## 4 Discussion

The present study aimed to highlight the plants used in Gabon to prevent and/or treat symptoms associated with COVID-19. Survey participants included individuals from the general population, traditional healers, and medicinal plant vendors. The findings revealed that most of the participants were male subjects, with the predominant age group being 31–44 years. These results suggest that men displayed a greater interest in the survey compared to women, likely due to the prevalence of male traditional healers and medicinal plant vendors. The age group of 31–44 years was particularly noteworthy as it represents the demographic most inclined to embrace traditional medicine and seek to reclaim traditional knowledge. In addition, a large number of informants had a university education. The high level of education among the majority of respondents indicates that, despite heightened awareness of the ongoing pandemic, the population continues to associate medicinal plants with the prevention and treatment of diseases exhibiting flu-like symptoms. Khadka et al. (2021) supported this finding, reporting that educated people had a preference for modern medicine, but during COVID-19, they used medicinal plants as an alternative medicine option. Gabon is very rich in plant biodiversity, and the knowledge of medicinal plants used to treat infections or conditions such as malaria or influenza is deeply rooted within the population, is transmitted from generation to generation, and is often shared by traditional healers with individuals seeking information. Consequently, when the pandemic emerged, the Gabonese population likened it to a blend of malaria and flu due to the overlapping symptoms with COVID-19, including fever, joint and body ache, fatigue, coughing, and cold symptoms. Subsequently, people began treating these symptoms using plants commonly used to treat influenza and malaria. The study findings indicate that these specific symptoms were the most frequently treated by the population. Surveys conducted in different countries such as Bangladesh and South Africa also described these symptoms as the most commonly treated in their respective countries (Phumthum et al., 2021; Rafiqul Islam et al., 2021; Zondi and Ehaine, 2022). The remedies predominantly involved the use of barks and leaves, prepared through infusion, decoction, or maceration. Notably, steam baths emerged as the second most common mode of administration, a method traditionally employed in Gabon specifically for treating malaria. Other studies also mentioned steam baths or steam inhalation as modes of administration, arguing that this method has been used for decades as a home remedy for cold and pain (Rafiqul Islam et al., 2021; Mvogo Ottou et al., 2022; Zondi and Ehaine, 2022). The study also revealed that the majority of respondents had contracted COVID-19 and that only 3% of them did not take treatment based on medicinal plants. Among the remaining respondents who claimed to have never developed any symptoms, medicinal plants were used for prevention. These results suggest that medicinal plants may have provided protection against coronavirus infection for individuals who used them. Further research, including clinical trials, is necessary to confirm these findings and to determine the safety and efficacy of the plants. In a similar context, results from a study carried out in Peru on the use of medicinal plants for COVID-19 prevention and respiratory symptom treatment showed that most of their respondents did use plants for these purposes (Villena-Tejada et al., 2021).

Ethnobotanical surveys often employ various indexes to unveil or highlight medicinal plants that are most frequently used or deemed most beneficial for the treatment of a specific disease. These indexes help quantify and analyze the importance of different plant species within a particular cultural or ecological context. In the present study, the results indicated that several indexes, including RFC, UV, or RI, collectively highlighted the plants most frequently used for the prevention or treatment of COVID-19. Notably, *Annickia chlorantha, Allium* sp.*, Citrus* sp.*, Alstonia congensis, Zingiber officinale, Carica papaya, Staudtia kamerunensis, Mangifera indica, Combretum micranthum, Cymbopogon citratus, Newbouldia laevis, Ocimum gratissimum, Gymnanthemum amygdalinum, Musa x paradisiaca,* and *Artemisia annua* emerged as the most commonly cited. Surveys conducted worldwide documented the use of several of these plants in the prevention and/or treatment of the disease, regardless of geographical location, climate diversity, or the variability of flora in the different regions. Hence, plants like *Allium* sp.*, Citrus* sp.*, Zingiber officinale, Carica papaya, Mentha piperita, Cymbopogon citratus*, and *Ocimum* sp. were used in diverse regions such as Cameroon, Morocco, Iraq, Thailand, Nepal, and Turkey (Akbulut, 2021; Khadka et al., 2021; Phumthum et al., 2021; Abdulrahman et al., 2022; Mvogo Ottou et al., 2022). ICF and POPUT are indispensable tools in ethnopharmacological surveys, offering valuable insights into the consensus, popularity, and cultural significance of medicinal plants within a community (Asiimwe et al., 2021; Anwar et al., 2023). Their application enhances the understanding of traditional healthcare practices and guides scientific research efforts. In this regard, symptoms such as fever, cough, and fatigue, which displayed high index values, appeared to be well-managed by the population, who are knowledgeable about several plants that can alleviate these symptoms. These plants merit further investigation based on the specific symptoms they are reported to alleviate.

The study findings suggest that plants traditionally used in the treatment of malaria are potential candidates for drug development against coronaviruses. Notably, the species prominently cited in this study have undergone assessment for their antiplasmodial activity, both in animal models and *in vitro*, demonstrating significant potential for managing malaria (Afolabi and Oyewole, 2020; Tajbakhsh et al., 2021; Indradi et al., 2023). These plants include *Annickia chlorantha*, *Zingiber officinale, Alstonia congensis, Newbouldia laevis, Ocimum gratissimum, Gymnanthemum amygdalinum, Artemisia annua*, and *Carica papaya* (Cimanga et al., 2019; Abubakar et al., 2020; Assogba, 2020; Wang et al., 2020; Kshirsagar and Rao, 2021; Tajbakhsh et al., 2021). Furthermore, the same plants demonstrate noteworthy anti-inflammatory and immunomodulatory effects, which are particularly valuable in addressing the cytokine storm induced by COVID-19 (Omoregie and Pal, 2016; Eftekhar et al., 2019; Abubakar et al., 2020; Olaoye et al., 2021; Kshirsagar and Rao, 2021; Mezui et al., 2022; Kamelnia et al., 2023; Nishitha et al., 2023; Ukwubile et al., 2023; Yuandani et al., 2023). In addition, plants such as *Z. officinale, Artemisia annua, Carica papaya*, *Citrus* sp., *Allium sativum,* and *Cymbopogon citratus* were assessed for their antiviral activity against SARS-CoV-2 (Belkessam et al., 2021; Haridas et al., 2021; Oladele et al., 2021; Thuy et al., 2021; Adel et al., 2022) (Table 4). Various studies, using molecular docking, cell-based assays, and clinical trials, were conducted to elucidate the mechanisms underlying the antiviral activity of the cited plants. Collectively, the findings of these studies suggest that all tested plants are potential putative inhibitors of the proliferation of SARS-CoV-2, ACE2 host receptor, and major protease. They impede the attachment, membrane fusion, and internalization of SARS-CoV-2 into host cells, as well as the viral replication and transcription processes. Furthermore, numerous trials currently investigate several promising and potent phyto-based formulations for the treatment of SARS-CoV-2 infections (Alam et al., 2021). These formulations include a range of bioactive metabolites, plant extracts, functional foods, and plant-based preparations. For example, hesperidin, which is present in some of the plants described in our study (Table 4), is involved as primary therapy in a phase II trial (Alam et al., 2021). In addition, a preliminary trial of the effect of *Allium sativum* in patients with SARS-CoV-2 showed an improvement in the general condition with the resolution of most of the symptoms (fever, headache, asthenia, ageusia, anosmia, and diarrhea) (Belkessam et al., 2021).

**TABLE 4:** Active compounds from medicinal plants with potential anti-SARS-CoV activity.

Plant name	Active compounds	Mechanisms	References
*Ageratum conyzoides*	Chromene, hydroxamic acid, and apigenin	Inhibit SARS-CoV-2 main protease	Hariono et al. (2021)
*Allium cepa*	Oleanolic acid, quercitrin, peonidin progesterone, and 3-arabinoside	Inhibit SARS-CoV-2 main protease	Fitriani and Utami (2021)
*Allium sativum*	Alliin, S-propyl cysteine, S-allylcysteine, squalene, S-ethylcysteine, 1,4-dihydro-2,3-benzoxathiin 3-oxide, 1,2,3-propanetriyl ester, trans-13-octadecenoic acid, and methyl-11-hexadecenoate Fresh bulbs	Inhibit SARS-CoV-2 6LU7 protein Improvement in the general condition with the resolution of most of the symptoms after 2 days	Pandey et al. (2021) Belkessam et al. (2021)
*Aloe vera*	Feralolide, isoaloeresin, aloeresine, and aloin A	Inhibit SARS-CoV-2 main protease	Mpiana, et al. (2020)
*Annona muricata*	Roseoside, coreximine Javoricin, 5-(1-hydroxytridecyl)oxolan-2-one, arianacin, annomuricin A, annomuricin B, annomuricin C, muricatocin C, muricatacin, *cis*-annonacin, annonacin-10-one, and *cis*-goniothalamicin	Inhibit the main protease and spike protein Inhibit the spike protein	Adedotun et al. (2023) Prasad et al. (2021)
*Artemisia annua*	Scopoletin, arteannuin, and artemisinic acid	Inhibit the main protease and spike protein	Baggieri et al. (2023)
*Capsicum chinense*	Kaempferol, lutein, zeaxanthin, and quercetin	Inhibit main protease, ACE-2, and TMPRSS2 proteins	Rahmattullah et al. (2021)
*Carica papaya*	Papain, β-cryptoxanthin, lycopene, lutein, β-carotene, dichloro-9,10-diphenylanthracene-9,10-diol, lupeol Deoxyquercetin, riboflavin, kaempferol, catechin, deoxykaempferol, and apigenin	Inhibitory activity against main proteases of SARS-CoV-2, SARS-CoV, and MERS-CoV Inhibit 3-chymotrypsin-like protease, papain-like protease, RNA-dependent RNA-polymerase, endonuclease, and S1 and S2 regions of the spike protein	Nallusamy et al. (2021) Hariyono et al. (2021)
*Cissus quadrangularis*	Taraxerol and β-amyrin	Inhibitory activity against main proteases of SARS-CoV-2, SARS-CoV, and MERS-CoV	Nallusamy et al. (2021)
*Citrus* sp.	Naringin, naringenin, hesperetin, hesperidin, nobiletin Obacunone, limonin, nomilin, hesperidin Sakuranetin, isosacuranetin, tetra-o-methylscutallerin, rutoside, eriodictoyl, quercetin, neoeriocitrin, diosmin, and diosmetin	Inhibit ACE2 receptor Virucidal activity on Vero E6-infected cells Inhibit SARS-CoV-2 main protease	Liu et al. (2022) Magurano et al. (2021) Maulydia et al. (2022) Khan et al. (2022)
*Cymbopogon citratus*	Tannic acid, isoorientin, luteolin, swertiajaponin, chlorogenic acid, cymbopogonol, warfarin, citral diethyl acetal, citral acetate, kaempferol, and cianidanol	Inhibit SARS-CoV-2 main protease	Ahmad et al. (2022)
*Gymnanthemum amygdalinum*	Veronicoside A, vernodalin and vernolide, vernomygdin and 11, 13-dihydrovernodalin, and neoandrographolide	Inhibit SARS-CoV-2 main protease	Oladele et al. (2021)
*Mangifera indica*	Ellagic acid, epicatechin, gallic acid, mangiferin, kaempferol Amentoflavone, catechin, mangiferin, and kaempferol	Inhibit SARS-CoV-2 main protease Inhibitory activity against main proteases of SARS-CoV-2, SARS-CoV, and MERS-CoV	Haruna et al. (2021) Nallusamy et al. (2021)
*Moringa oleifera*	Catechin, ellagic acid, chlorogenic acid, quercetin, Myrecitin, and kaempferol Epicatechin, niazirin, glucotropaeolin, quercetin, apigenin, luteolin, rutin, kaempferol, isorhamnetin, myricetin, astragalin, marumoside A, and moringyne	Inhibit SARS-CoV-2 main protease Inhibit the human TMPRSS2 protein	Haruna et al. (2021) Oyedara et al. (2021)
*Musa x paradisiaca*	Leucocyanidin, quercetin, sitoindoside-I, 6S-9R-roseoside, hydroxyanigorufone, and 1,2-dihydro-1,2,3-trihydroxy-9-[4 methyphenylphenalene]	Inhibit SARS-CoV-2 main protease	Harini and Gopal (2022)
*Ocimum gratissimum*	Luteolin, rosmarinic acid, chicoric acid, and myricetin	Inhibit SARS-CoV-2 main protease	Gyebi et al. (2021)
*Psidium guajava*	Gamma-sitosterol, peri-xanthenoxanthene-4,10-dione,2,8-bis (1-methylethyl) *P. guajava* extract supplementation	Inhibit main protease, papain-like protease, and spike and ACE2 receptor. Neutrophil/lymphocyte ratio reduction, PCR-based conversion time acceleration, and increase in the recovery rate of subjects with mild and asymptomatic COVID-19 infection in a single-blinded, randomized clinical trial	Fadilah et al. (2021) Heppy et al. (2023)
*Pycnanthus angolensis*	*Pycnanthuquinone* *C* and *pycnanthuquinone A*	Inhibit SARS-CoV-2 main protease	Chtita et al. (2022)
*Syzygium aromaticum*	Campesterol, stigmasterol, crategolic acid, oleanolic acid, and bicornin Polysaccharides	Inhibit SARS-CoV-2 main protease Block SARS-CoV-2 replication	Abdelli and Hamed (2023) Jin et al. (2021)
*Xylopia aethiopica*	Phenolic compounds and essential oils Liriodenine, lysicamine, o-methylmoschatoline, oxoglaucine, and oxophoebine	Antiviral activity against SARS-CoV-1 and SARS-CoV-2 pseudoviruses infecting HeLa ACE-2 cells Inhibit SARS-CoV-2 main protease	Melo et al. (2021) Ogunyemi and Oderinlo (2022)
*Zingiber officinale*	Gingerenone-A, chlorogenic acid, and hesperidin Cyanin Thujopsene, zingiberol, Gamma-elemene, beta-elemene, and aromadendrene	Block the entry of SARS-CoV-2 through its ACE2 receptors, binding affinities to Mpro and S protein Inhibitory activity against main proteases of SARS-CoV-2, SARS-CoV, and MERS-CoV Inhibit human TMPRSS2 protein	Jahan et al. (2021) Nallusamy et al. (2021) Ogunyemi and Oderinlo (2022)

Analysis of the data highlighted that all the plants described in Table 4 showing promising activity against SARS-CoV-2 are listed in Table 3 as components of the popular recipes used in the prevention and/or treatment of COVID-19. A combination of these plants with antiviral and anti-inflammatory effects in a recipe strengthens the hypothesis that these recipes could effectively prevent or treat the coronavirus infection, thereby sustaining their use by the Gabonese population. So, taken together, the results of the present study showed the potential of medicinal plants as independent therapies, complementary or alternative medicines for the management of symptoms associated with COVID-19.

A concern could be raised regarding the potential overharvesting of certain species such as *Annickia chlorantha, Allium* sp.*, Citrus* sp.*, Alstonia congensis, Zingiber officinale, Carica papaya, Staudtia kamerunensis, Mangifera indica, Combretum micranthum,* and *Cymbopogon citratus* due to increased demand during the pandemic. However, Gabon is predominantly covered by forest (over 80%), and most of these species are distributed across all regions of the country (Wakefield Adhya, 2024). This suggests that the overharvesting of these species during the pandemic may not have had a significant impact on their abundance. Nevertheless, it is important to develop conservation planning for species with significant bioactivity. Conservation efforts could include measures such as sustainable harvesting practices and community-based management initiatives to ensure the long-term viability of these species and their ecosystems.

### 4.1 Limitations

The online ethnobotanical survey fell short of the anticipated participant count, primarily attributed to participants’ failure to share the survey link and a general lack of interest in responding to online surveys. Furthermore, the use of online surveys is not common among the Gabonese population, resulting in responders who are likely to possess a certain level of education and understand the significance of the survey. This could lead to potential bias regarding the sociodemographic profile of the responders. Furthermore, a field-based study extended to all the regions of the country might cover responses from all levels and classes of people.

## 5 Conclusion

COVID-19 has emerged as the most prolonged and deadly coronavirus outbreak witnessed worldwide in the past 50 years. Despite extensive exploration of various treatments, no definitive solution has been identified, and vaccination efforts have encountered limitations in the face of evolving virus variants. Ethnobotanical surveys conducted worldwide have explored the potential of plants to alleviate COVID-19 symptoms, yielding positive results and encouraging the use of medicinal plants for coronavirus infection management. In Gabon, the country’s relatively low rate of cases and fatalities has been attributed to the consumption of plants traditionally used to treat malaria and flu, both as a preventive measure and in the treatment of COVID-19. Further investigation into the mechanisms of these plants such as anti-inflammatory, antioxidant, antiviral, and immunomodulatory activities is crucial for the development of plant-based medicines that could effectively act during the early stages of SARS-CoV-2 infection. This research holds promise as a significant alternative in the preparation for the inevitable occurrence of future coronavirus epidemics in the coming years. Understanding and harnessing the potential of these medicinal plants may provide valuable tools for mitigating the impact of such outbreaks.

## Data Availability

The original contributions presented in the study are included in the article/Supplementary Material; further inquiries can be directed to the corresponding author.
